# Supplementary material for: Modeling glioblastoma heterogeneity as a dynamic network of cell states
Source: Mol Syst Biol. 2021 Sep 16;17(9):e10105. doi: 10.15252/msb.202010105 (PMC8444284; doi:10.15252/msb.202010105)
Supplement: Supplementary file 6 — Source Data for Figure 5 [file MSB-17-e10105-s004.zip › Figure5A_sourcedata/GSEA_3071/hallmarks_stateII.GseaPreranked.1623742536692/HALLMARK_MTORC1_SIGNALING.html]

Details for gene set HALLMARK\_MTORC1\_SIGNALING[GSEA]

|  || Dataset | state23071 |
| Phenotype | NoPhenotypeAvailable |
| Upregulated in class | na\_pos |
| GeneSet | HALLMARK\_MTORC1\_SIGNALING |
| Enrichment Score (ES) | 0.2984598 |
| Normalized Enrichment Score (NES) | 1.8032285 |
| Nominal p-value | 0.0 |
| FDR q-value | 0.012352426 |
| FWER p-Value | 0.094 |
Table: GSEA Results Summary

  

Fig 1: Enrichment plot: HALLMARK\_MTORC1\_SIGNALING      
 Profile of the Running ES Score & Positions of GeneSet Members on the Rank Ordered List

  

| PROBE | GENE SYMBOL | GENE\_TITLE | RANK IN GENE LIST | RANK METRIC SCORE | RUNNING ES | CORE ENRICHMENT || 1 | RRM2 |  |  | 3 | 1.857 | 0.0359 | Yes |
| 2 | MCM4 |  |  | 81 | 1.110 | 0.0386 | Yes |
| 3 | DHFR |  |  | 101 | 1.051 | 0.0546 | Yes |
| 4 | TUBA4A |  |  | 246 | 0.827 | 0.0349 | Yes |
| 5 | CCNF |  |  | 321 | 0.737 | 0.0310 | Yes |
| 6 | EBP |  |  | 336 | 0.721 | 0.0417 | Yes |
| 7 | MTHFD2 |  |  | 337 | 0.721 | 0.0560 | Yes |
| 8 | RPA1 |  |  | 343 | 0.715 | 0.0688 | Yes |
| 9 | HPRT1 |  |  | 351 | 0.709 | 0.0811 | Yes |
| 10 | UCHL5 |  |  | 354 | 0.708 | 0.0946 | Yes |
| 11 | TOMM40 |  |  | 358 | 0.706 | 0.1078 | Yes |
| 12 | AURKA |  |  | 360 | 0.705 | 0.1215 | Yes |
| 13 | PSMG1 |  |  | 372 | 0.696 | 0.1325 | Yes |
| 14 | TMEM97 |  |  | 403 | 0.676 | 0.1383 | Yes |
| 15 | PHGDH |  |  | 429 | 0.664 | 0.1452 | Yes |
| 16 | PNP |  |  | 434 | 0.659 | 0.1572 | Yes |
| 17 | ARPC5L |  |  | 471 | 0.640 | 0.1609 | Yes |
| 18 | ITGB2 |  |  | 476 | 0.639 | 0.1725 | Yes |
| 19 | SLC9A3R1 |  |  | 497 | 0.630 | 0.1799 | Yes |
| 20 | FADS1 |  |  | 498 | 0.629 | 0.1924 | Yes |
| 21 | ENO1 |  |  | 501 | 0.629 | 0.2043 | Yes |
| 22 | DDX39A |  |  | 542 | 0.605 | 0.2062 | Yes |
| 23 | CDC25A |  |  | 572 | 0.589 | 0.2106 | Yes |
| 24 | HSPD1 |  |  | 606 | 0.576 | 0.2137 | Yes |
| 25 | CACYBP |  |  | 613 | 0.573 | 0.2235 | Yes |
| 26 | TUBG1 |  |  | 617 | 0.570 | 0.2340 | Yes |
| 27 | NUP205 |  |  | 705 | 0.531 | 0.2228 | Yes |
| 28 | CCT6A |  |  | 725 | 0.525 | 0.2284 | Yes |
| 29 | MCM2 |  |  | 734 | 0.521 | 0.2367 | Yes |
| 30 | UNG |  |  | 750 | 0.516 | 0.2431 | Yes |
| 31 | SLC1A5 |  |  | 757 | 0.514 | 0.2518 | Yes |
| 32 | ERO1A |  |  | 767 | 0.510 | 0.2596 | Yes |
| 33 | SLC2A1 |  |  | 787 | 0.503 | 0.2648 | Yes |
| 34 | CTSC |  |  | 833 | 0.489 | 0.2632 | Yes |
| 35 | ME1 |  |  | 835 | 0.489 | 0.2726 | Yes |
| 36 | EEF1E1 |  |  | 840 | 0.488 | 0.2812 | Yes |
| 37 | ACTR3 |  |  | 909 | 0.469 | 0.2735 | Yes |
| 38 | CYP51A1 |  |  | 952 | 0.458 | 0.2720 | Yes |
| 39 | SLC7A11 |  |  | 998 | 0.445 | 0.2696 | Yes |
| 40 | PLK1 |  |  | 1009 | 0.442 | 0.2758 | Yes |
| 41 | SEC11A |  |  | 1019 | 0.438 | 0.2822 | Yes |
| 42 | CDKN1A |  |  | 1088 | 0.419 | 0.2735 | Yes |
| 43 | TPI1 |  |  | 1111 | 0.415 | 0.2762 | Yes |
| 44 | GMPS |  |  | 1126 | 0.413 | 0.2809 | Yes |
| 45 | MAP2K3 |  |  | 1136 | 0.410 | 0.2867 | Yes |
| 46 | BUB1 |  |  | 1145 | 0.408 | 0.2928 | Yes |
| 47 | STIP1 |  |  | 1227 | 0.392 | 0.2803 | Yes |
| 48 | DHCR24 |  |  | 1253 | 0.386 | 0.2816 | Yes |
| 49 | TXNRD1 |  |  | 1269 | 0.383 | 0.2855 | Yes |
| 50 | PPP1R15A |  |  | 1284 | 0.381 | 0.2895 | Yes |
| 51 | G6PD |  |  | 1346 | 0.368 | 0.2815 | Yes |
| 52 | SHMT2 |  |  | 1361 | 0.366 | 0.2852 | Yes |
| 53 | LDHA |  |  | 1374 | 0.361 | 0.2894 | Yes |
| 54 | SLC7A5 |  |  | 1434 | 0.351 | 0.2815 | Yes |
| 55 | IMMT |  |  | 1436 | 0.350 | 0.2882 | Yes |
| 56 | POLR3G |  |  | 1457 | 0.346 | 0.2900 | Yes |
| 57 | CYB5B |  |  | 1491 | 0.342 | 0.2886 | Yes |
| 58 | PSAT1 |  |  | 1505 | 0.340 | 0.2920 | Yes |
| 59 | ETF1 |  |  | 1507 | 0.339 | 0.2985 | Yes |
| 60 | PSMA3 |  |  | 1692 | 0.312 | 0.2586 | No |
| 61 | ATP5MC1 |  |  | 1769 | 0.302 | 0.2456 | No |
| 62 | PITPNB |  |  | 1776 | 0.302 | 0.2500 | No |
| 63 | HSPA9 |  |  | 1884 | 0.287 | 0.2290 | No |
| 64 | PNO1 |  |  | 1888 | 0.287 | 0.2339 | No |
| 65 | SDF2L1 |  |  | 1936 | 0.281 | 0.2277 | No |
| 66 | ATP6V1D |  |  | 1963 | 0.277 | 0.2267 | No |
| 67 | XBP1 |  |  | 1995 | 0.273 | 0.2243 | No |
| 68 | RRP9 |  |  | 2055 | 0.267 | 0.2148 | No |
| 69 | NFIL3 |  |  | 2056 | 0.267 | 0.2201 | No |
| 70 | PPIA |  |  | 2072 | 0.265 | 0.2216 | No |
| 71 | SORD |  |  | 2077 | 0.265 | 0.2258 | No |
| 72 | PGK1 |  |  | 2126 | 0.260 | 0.2189 | No |
| 73 | LDLR |  |  | 2128 | 0.260 | 0.2238 | No |
| 74 | SERPINH1 |  |  | 2146 | 0.258 | 0.2247 | No |
| 75 | PSME3 |  |  | 2207 | 0.251 | 0.2146 | No |
| 76 | GTF2H1 |  |  | 2211 | 0.251 | 0.2188 | No |
| 77 | ACSL3 |  |  | 2288 | -0.258 | 0.2049 | No |
| 78 | FADS2 |  |  | 2338 | -0.263 | 0.1979 | No |
| 79 | CANX |  |  | 2427 | -0.274 | 0.1813 | No |
| 80 | SKAP2 |  |  | 2581 | -0.298 | 0.1489 | No |
| 81 | RIT1 |  |  | 2594 | -0.300 | 0.1518 | No |
| 82 | GSK3B |  |  | 2641 | -0.307 | 0.1464 | No |
| 83 | HSP90B1 |  |  | 2648 | -0.308 | 0.1510 | No |
| 84 | MLLT11 |  |  | 2760 | -0.328 | 0.1297 | No |
| 85 | IGFBP5 |  |  | 2820 | -0.339 | 0.1217 | No |
| 86 | GLRX |  |  | 2834 | -0.342 | 0.1252 | No |
| 87 | ELOVL6 |  |  | 2958 | -0.366 | 0.1016 | No |
| 88 | SQSTM1 |  |  | 3013 | -0.378 | 0.0956 | No |
| 89 | MTHFD2L |  |  | 3105 | -0.401 | 0.0808 | No |
| 90 | PIK3R3 |  |  | 3151 | -0.414 | 0.0777 | No |
| 91 | HSPA5 |  |  | 3335 | -0.468 | 0.0412 | No |
| 92 | CD9 |  |  | 3567 | -0.581 | -0.0051 | No |
| 93 | CXCR4 |  |  | 3733 | -0.710 | -0.0323 | No |
| 94 | NUPR1 |  |  | 3825 | -0.813 | -0.0390 | No |
| 95 | IDH1 |  |  | 3842 | -0.832 | -0.0266 | No |
| 96 | BTG2 |  |  | 3917 | -0.999 | -0.0253 | No |
| 97 | TM7SF2 |  |  | 3918 | -0.999 | -0.0056 | No |
| 98 | CTH |  |  | 3970 | -1.112 | 0.0036 | No |
| 99 | STC1 |  |  | 4026 | -1.424 | 0.0180 | No |
Table: GSEA details [plain text format]

  

Fig 2: HALLMARK\_MTORC1\_SIGNALING: Random ES distribution      
 Gene set null distribution of ES for **HALLMARK\_MTORC1\_SIGNALING**
